# Generation of a Jurkat-based fluorescent reporter cell line to evaluate lipid antigen interaction with the human iNKT cell receptor

**DOI:** 10.1038/s41598-019-43529-4

**Published:** 2019-05-15

**Authors:** Piotr Humeniuk, Sabine Geiselhart, Claire Battin, Tonya Webb, Peter Steinberger, Wolfgang Paster, Karin Hoffmann-Sommergruber

**Affiliations:** 1 Department of Pathophysiology and Allergy Research, Medical University of Vienna, Vienna, Austria; 2 Institute of Immunology, Division of Immune Receptors and T cell Activation, Medical University of Vienna, Vienna, Austria; 3 Department of Microbiology & Immunology, University of Maryland School of Medicine, Baltimore, USA; 4 Present Address: Children’s Cancer Research Institute, St. Anna Kinderkrebsforschung, Vienna, Austria

**Keywords:** Immunology, Lymphocytes

## Abstract

Invariant natural killer T (iNKT) cells are a specialized subset of T cells contributing to both, the innate and adaptive immune responses. In contrast to conventional T lymphocytes they recognize lipid antigens. The aim of the project is to establish a novel model system, to study iNKT-TCR – ligand interaction. An iNKT reporter cell line (JE6-1^REP-iNKT^) was engineered by introducing the human iNKT-TCR into a human leukemic T cell line carrying an NF-κB-driven fluorescent transcriptional reporter construct. Antigen presenting BW^STIM^ cells expressing human CD1d and CD80 were generated. Reporter induction in JE6-1^REP-iNKT^ cells was assessed by flow cytometry. CRISPR/Cas9 was used for β2M knock out in JE6-1^REP-iNKT^ cells to abrogate CD1d expression and thus excluding antigen self-presentation. Reporter cells were shown to specifically react with iNKT antigens presented via CD1d. Their sensitivity towards α-GalCer was comparable to a murine iNKT hybridoma cell line. In conclusion, we created a novel iNKT reporter platform which, compared to traditional iNKT cell assays, is characterized by a shorter turnaround time and lower costs. It thus facilitates the identification of antigenic structures that drive the activation of iNKT cells in health and disease.

## Introduction

Natural killer T cells expressing an invariant T cell receptor α-chain (iNKT cells) are a unique and evolutionary conserved subset of lymphocytes. In contrast to conventional T lymphocytes, which recognize peptide antigens in the context of major histocompatibility complex (MHC) molecules, iNKT cells recognize lipid antigens presented by CD1d, a non-polymorphic MHC class I homolog. CD1d is expressed on many types of epithelial and endothelial cells and professional antigen-presenting cells. Invariant NKT cells exhibit characteristics of innate and adaptive immune cells and upon activation not only display killer cell activity, but also rapidly secrete large amounts of cytokines. Both, protective and pathological roles have been ascribed to iNKT cells.

In murine iNKT cells, the invariant Vα14-Jα18 TCRα chain is paired with a restricted repertoire of β chains, including Vβ2, Vβ7 and most commonly Vβ8.2, which is expressed in approximately 60% of murine iNKT cells^1,2^. In C57BL/6 wild-type mice, iNKT cells are most abundant in the liver, where they represent around 22% of mononuclear cells. Lower percentages were observed in the thymus, spleen and bone marrow. In murine peripheral blood, iNKT cells constitute ~0.5% of mononuclear cells^3^. To facilitate research on murine iNKT cells, hybridoma cell lines were successfully generated and have proven invaluable tools to study the biology of iNKT cells^4–7^. In human iNKT cells, the invariant Vα24-Jα18 TCRα chain is paired with the Vβ11 TCRβ chain^8^. In humans, the frequency of iNKT cells is usually much lower than in mice and a high degree of variability in iNKT cell numbers between individuals has been reported. In healthy individuals approximately 0.01–1% of peripheral blood mononuclear cells (PBMCs) are iNKT cells^9^.

Upon activation, iNKT cells can significantly affect immune responses by promoting the secretion of Th1, Th2 or Th17 immune regulatory cytokine patterns. Invariant NKT cells have been identified as important players in health and disease^10,11^. Several studies implicated iNKT cells in immune responses against tumors^12^. A beneficial role of iNKT cells was further reported in several bacterial and viral infections^13^. For example, in a mouse model for Lyme disease caused by *Borrelia burgdorferi*, iNKT cells showed a protective role during infection by inhibiting pathogen spreading^14^. Pulmonary infections with *Streptococcus pneumoniae*^15^, *Pseudomonas aeruginosa*^16^, and influenza virus^17^ are further examples where iNKT cells activated via CD1d antigen presentation, expand and provide the cytokine milieu to attract neutrophils and macrophages. Additionally, the importance of iNKT cells has been described in the context of obesity^18^ and insulin resistance^19^. Recent findings indicate the participation of iNKT cells in the pathomechanisms of allergy and asthma^20,21^. However, whether they generally play a protective or pathological role is still under debate^13^.

Antigens for iNKT cells are present in the environment^22^. The first described iNKT cell antigen, α-galactosylceramide (α-GalCer), was originally purified from the deep sea sponge *Agelas mauritanius* and identified through its strong anti-tumor properties in mice^23^. Binding of the lipid antigen to CD1d occurs via the two lipid chains, exposing the α-linked sugar group for recognition by the invariant TCR. Although the majority of mammalian glycolipids utilize a β-linkage of the sugar^24^, most antigenic ceramide lipids show α-anomeric linkage of a galactose sugar which is crucial for its stimulatory capacity^23,25^. These lipid antigens vary in their lipid tails as well as in their head group and show different affinities for both, CD1d and the TCR^26^ and thus determining Th1/Th2 balance^27,28^. Therefore, the identification of new lipid antigens is highly relevant for developing iNKT cell based therapies. Stimulation of iNKT cells with α-GalCer causes rapid production of Th1 and Th2 cytokines, including IL-4, IL-10, IL-13 and IFN-γ, thus strongly enhancing immune responses. Anti-cancer properties of α-GalCer have been tested in several preclinical and clinical studies, which showed promising results^29–31^. The sphingosine truncated derivative of α-GalCer, OCH, is characterized by lower CD1d and iNKT-TCR affinity, and preferentially stimulates iNKT cells to produce Th2 cytokines. In a murine model of experimental autoimmune encephalomyelitis (EAE), OCH showed protective properties superior to α-GalCer^32^. 7DW8-5, a recently characterized analog of α-GalCer with strongly increased CD1d affinity, is characterized by a fluorinated benzene ring at the end of a shorter C8 length fatty acyl chain. 7DW8-5 shows significantly increased biological activity and was designed as a potential malaria and HIV vaccine adjuvant^26^.

The small number of iNKT cells in human peripheral blood and the lack of human iNKT hybridoma cell lines makes it challenging to study these cells. Reliable methods of iNKT cell expansion using artificial antigen presenting cells already exist, however they are time consuming and technically complex^33^.

To overcome the difficulties in studying human iNKT cells, we aimed to generate a fluorescence-based human iNKT-TCR reporter system, applicable for screening potential new lipid antigens that influence iNKT cell activation.

## Results

### Generation of an iNKT-TCR-transgenic reporter T cell line

**Figure 1:**
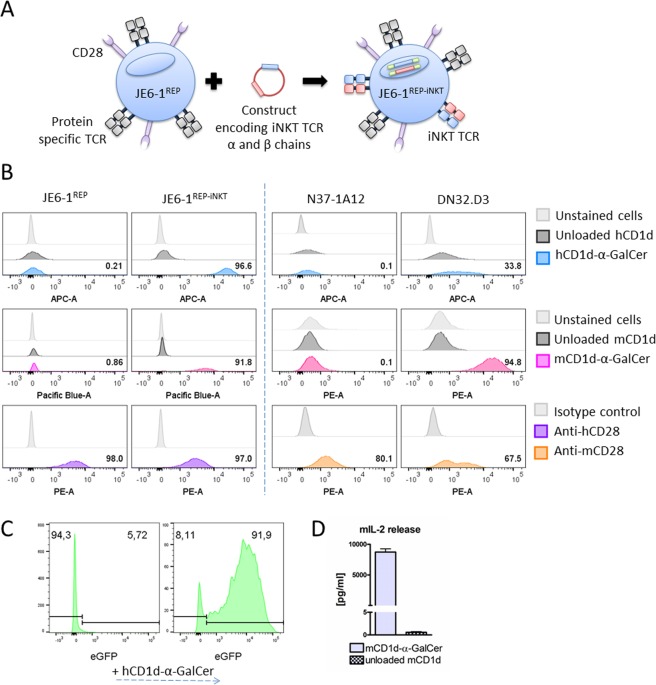
Generation of an iNKT-TCR-transgenic reporter T cell line. (**A**) JE6.1-NF-κB::eGFP iNKT (JE6-1^REP-iNKT^) cells were generated by transducing the parental JE6.1-NF-κB::eGFP (JE6-1^REP^) with the alpha and beta chains of the human iNKT-TCR^36^. (**B**) Surface staining of JE6-1^REP^, JE6-1^REP-iNKT^ and murine hybridoma DN32.D3 and N37-1A12 cells was performed with recombinant human (upper panel) and murine (middle panel) α-GalCer loaded CD1d and human and murine anti-CD28 antibodies (lower panel). Numbers show the percentages of positive cells. (**C**) eGFP expression of JE6-1^REP-iNKT^ cells before and after activation with α-GalCer loaded plate immobilized hCD1d (10 µg/ml). (**D**) Release of IL-2 from DN32.D3 stimulated with immobilized α-GalCer loaded versus unloaded mCD1d (10 µg/ml).

We have recently described Jurkat E6.1 NF-κB::eGFP, a highly sensitive reporter T cell line allowing fluorescence-based readout of NF-κB transcriptional activity^34,35^. For the current study, Jurkat E6.1 NF-κB::eGFP were transduced with the human iNKT-TCR (TCR V-alpha chain: GenBank - ABC72374.1; TCR V-beta chain: GenBank - EAW51929.1) and designated JE6-1^REP-iNKT^ (Fig. 1A)^36^. 2A-peptide mediated co-expression of the iNKT-TCR α and β chains with the puromycin N-acetyl-transferase allowed efficient selection of cells containing the iNKT-TCR encoding construct. Surface expression of the iNKT-TCR in the JE6-1^REP-iNKT^ reporter cell line was verified by positive staining with APC-labelled α-GalCer loaded CD1d tetramers (Fig. 1B, upper panel). Parental JE6-1^REP^ cells lack the iNKT-TCR and as expected no specific signal with APC-labelled α-GalCer loaded CD1d tetramers was detected. Due to highly conserved sequences between murine and human CD1d, a strong cross-reactivity was observed when testing our reporter cell line with murine CD1d dextramers loaded with α-GalCer (Fig. 1B, middle panel). CD28 expression was confirmed for both, parental and iNKT-TCR-transduced reporters (Fig. 1B, lower panel). The well-established murine hybridoma cell line DN32.D3^37^ stained positive for both, the iNKT-TCR and CD28 (Fig. 1B – right panel), in contrast to the control cell line N37-1A12^38^, which stained positive only for CD28.

For functional testing, the reporter cells were cultivated on plate-bound CD1d loaded with α-GalCer. Over-night incubation led to robust NF-κB-driven eGFP expression, with >90% of positive cells (Fig. 1C). DN32.D3 cells cultured on similarly prepared α-GalCer-loaded CD1d surfaces responded by IL-2 production, which could be measured in the supernatant (Fig. 1D).

### JE6-1^REP-iNKT^ display dose-dependent transcriptional reporter activation in response to α-GalCer-loaded CD1d

**Figure 2:**
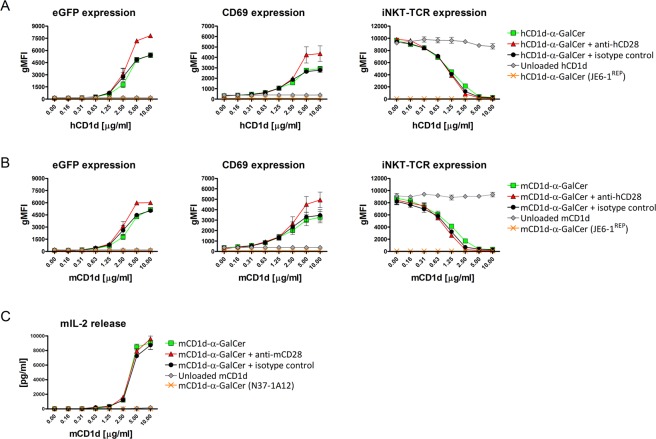
Activation of JE6-1^REP-iNKT^ cells. JE6-1^REP-iNKT^ cells were stimulated with immobilized α-GalCer loaded human (**A**) and murine (**B**) CD1d alone, or in combination with a soluble human CD28 antibody. Unloaded CD1d served as control. Following 24 h of stimulation, reporter gene expression, CD69 upregulation and iNKT receptor downregulation on reporter cells were measured by flow cytometry. Results are presented as geometric mean of fluorescence intensity (gMFI). (**C**) Murine hybridoma cells (DN32.D3) were stimulated analogically as our reporters. The soluble murine CD28 antibody provided co-stimulation. Activation was assessed by measuring the IL-2 release. The murine hybridoma cell line N37-1A12 was used as a negative control. gMFI and IL-2 concentrations (pg/ml) are shown for three independent experiments performed in triplicates.

We first tested the sensitivity of our JE6-1^REP-iNKT^ reporter cells in an *in vitro* assay. Following overnight incubation with α-GalCer loaded human CD1d immobilized on culture plates, our reporter cells responded in a dose-dependent manner, starting at a concentration of 0.63 µg/ml CD1d (Fig. 2A, left panel). Providing a co-stimulatory signal via a soluble monoclonal CD28 antibody resulted in increased eGFP-levels, but did not enhance sensitivity. In addition, activation of the JE6-1^REP-iNKT^ cells was assessed via the surface expression of the early activation marker CD69 (Fig. 2A, middle panel). Upregulation of CD69 could be detected starting at a concentration of 0.63 µg/ml CD1d. Again, co-stimulation via CD28 resulted in an almost two-fold increase of CD69 expression while the sensitivity of the system remained unchanged. During this experiment, expression of the iNKT-TCR was assessed. Surface expression of the iNKT-TCR decreased in a dose-dependent manner starting already at a concentration of 0.31 µg/ml of loaded CD1d and was not detectable at concentrations of 5 µg/ml and higher (Fig. 2A, right panel). Next, the activation of our reporter cell system was tested using α-GalCer loaded murine CD1d in order to compare cross reactivity (Fig. 2B). Response to mouse CD1d complexes was comparable due to highly conserved sequences between human and mouse CD1d proteins. Co-stimulation via CD28 increased the signal of eGFP and CD69 expression. Again downregulation of surface iNKT-TCR expression was observed, starting at 0.31 µg/ml of loaded murine CD1d (Fig. 2B, left panel). As a reference, the murine hybridoma cell line DN32.D3 was tested, using IL-2 release as readout (Fig. 2C). The sensitivity of DN32.D3 was comparable to our reporter cell system with activation starting at 0.63 µg/ml of loaded CD1d. Addition of a murine CD28 antibody did not affect the response of DN32.D3 cells. Our data demonstrate that JE6-1^REP-iNKT^ cells are equally sensitive as the commonly used DN32.D3 hybridoma cell line.

### Generation of human CD1d-expressing BW-based stimulator cells

**Figure 3:**
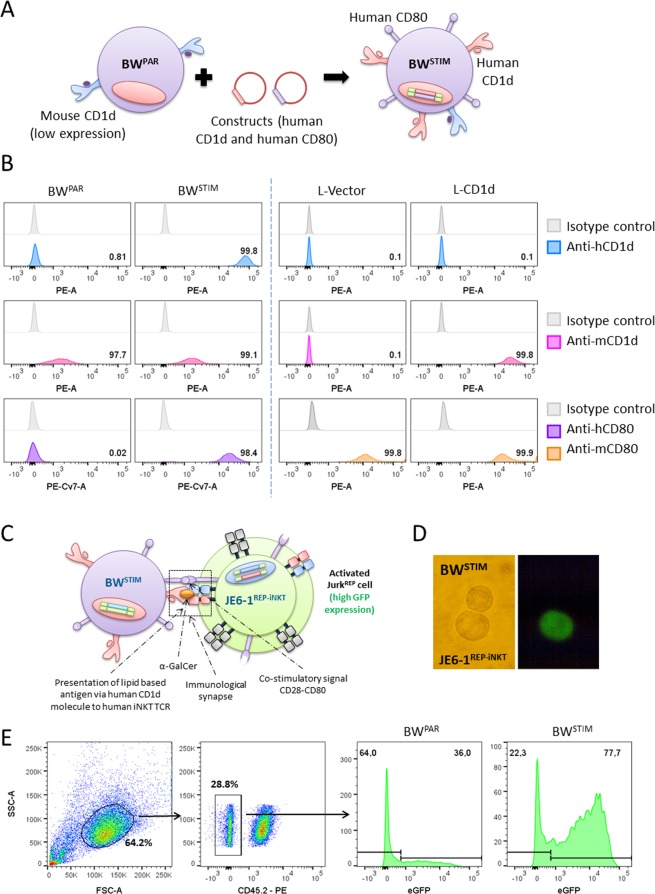
Generation of a BW-based antigen presenting cell line. (**A**) BW parental cells (BW^PAR^) were transduced with human CD1d and CD80 to generate antigen presenting stimulatory cells (BW^STIM^). (**B**) Surface expression analysis of CD1d and CD80 by flow cytometry. Numbers indicate the percentage of positive cells. (**C**) Principle of reporter cell stimulation. (**D**) Fluorescence microscopy image of an activated reporter cell. (**E**) Gating strategy to analyze activation levels of JE6-1^REP-iNKT^ cells. Murine CD45 expression was used to exclude BW^STIM^ cells during analysis. Histograms represent eGFP expression after co-culture experiments of JE6-1^REP-iNKT^ with BW^PAR^ (left) and BW^STIM^ (right) cells in the presence of α-GalCer (5 ng/ml).

For cellular activation assays, DN32.D3 hybridoma cells are typically incubated with lipid-loaded CD1d. To provide a more physiological stimulation, particularly for lipid antigens that require processing, a cellular CD1d-lipid presentation system would be advantageous. To this end, we introduced human CD1d and CD80 into the murine thymoma cell line BW5147 (BW). A single high expression cell clone was chosen for further experiments designated BW^STIM^ (Fig. 3A). BW cells are rapidly dividing suspension cells. As such they are a convenient, easy-to-use, antigen presenting cell line. Additionally, L-CD1d cells (CD1d transfected murine fibroblasts)^39^ were analyzed for their surface expression of CD1d and CD80.

Constitutive low-level expression of murine CD1d was observed on BW cells (Fig. 3B, left panel). L-CD1d cells were highly positive for murine CD1d and CD80 and negative for human CD1d, respectively (Fig. 3B, right panel). L-vector cells (used as a control) were negative for both, human and murine CD1d and positive for murine CD80. Lipid antigen presentation by BW^STIM^ cells was assessed by co-culture with JE6-1^REP-iNKT^ cells in the presence of α-GalCer (Fig. 3C). Fluorescence microscopy of an activated reporter cell (Fig. 3D) and flow cytometry analysis of eGFP expression (Fig. 3E) demonstrated that the generated BW^STIM^ cell line has a strong capacity to present lipid antigens.

### JE6-1^REP-iNKT^ cell activation in co-culture assays with BW^STIM^ and L-CD1d cells

**Figure 4:**
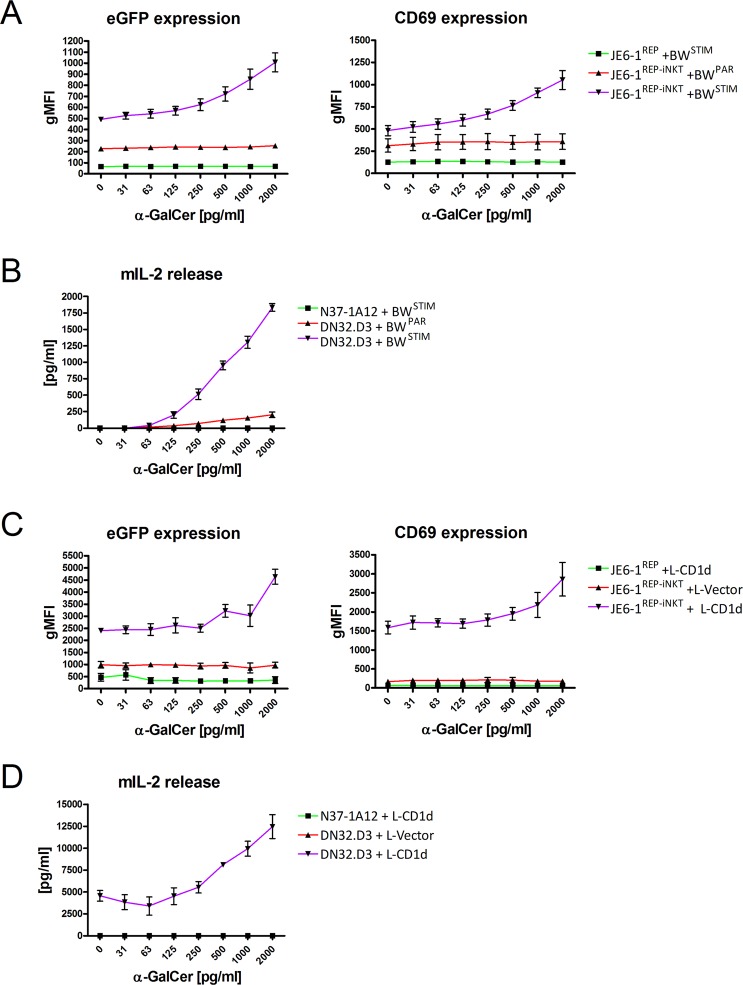
Activation of JE6-1^REP-iNKT^ cells in co-culture assays. (**A**) Co-culture experiments of JE6-1^REP-iNKT^ with α-GalCer pulsed murine BW^STIM^ cells (parental BW^PAR^ and JE6-1^REP^ served as controls). (**B**) Murine hybridoma (DN32.D3, N37-1A12) cells were activated in a similar fashion as in A. IL-2 release was used as activation marker. (**C**) Co-culture experiments of JE6-1^REP-iNKT^ with murine L-CD1d cells (again, JE6-1^REP^ and L-Vector cells served as controls). NF-κB-driven eGFP expression (left) and CD69 upregulation (right) were assessed by flow cytometry. (**D**) Murine hybridoma (DN32.D3, N37-1A12) cells were activated in a similar fashion as in (C). IL-2 release was used as activation marker. Three independent experiments performed in triplicates are shown.

Further, we tested our reporter cell line in co-culture with BW^STIM^ cells. Again, α-GalCer was used as lipid antigen and eGFP and CD69 expression as activation markers. Increasing concentrations of α-GalCer resulted in a dose-dependent stimulation of the reporter cells, starting at a concentration of 31 pg/ml α-GalCer (Fig. 4A) and continuously increasing up to 2,000 pg/ml without reaching a plateau. Control cultures (JE6-1^REP-iNKT^/BW^PAR^ or JE6-1^REP^/BW^STIM^) did not induce cellular activation. In parallel, co-culture experiments of our stimulator cells BW^STIM^ and the reference cell line DN32.D3 were performed. As already observed for plate-bound CD1d, the sensitivity was comparable to JE6-1^REP-iNKT^ reporter cells (Fig. 4B). Alternatively, JE6-1^REP-iNKT^ cells were cultured with L-CD1d cells. As shown in Fig. 4C-D, L-CD1d cells endogenously expressing an activating antigen are able to stimulate both, JE6-1^REP-iNKT^ reporter and DN32.D3 cells, respectively. Addition of α-GalCer resulted in a dose-dependent increase of eGFP and CD69 expression starting at a concentration of 500 pg/ml. L-vector cells, used as a control, did not show an increase of eGFP and CD69 expression after addition of α-GalCer. Again, DN32.D3 hybridoma cells showed a similar sensitivity as demonstrated by increased IL-2 release.

### Genetic ablation of β2 microglobulin eliminates antigen self-presentation

**Figure 5:**
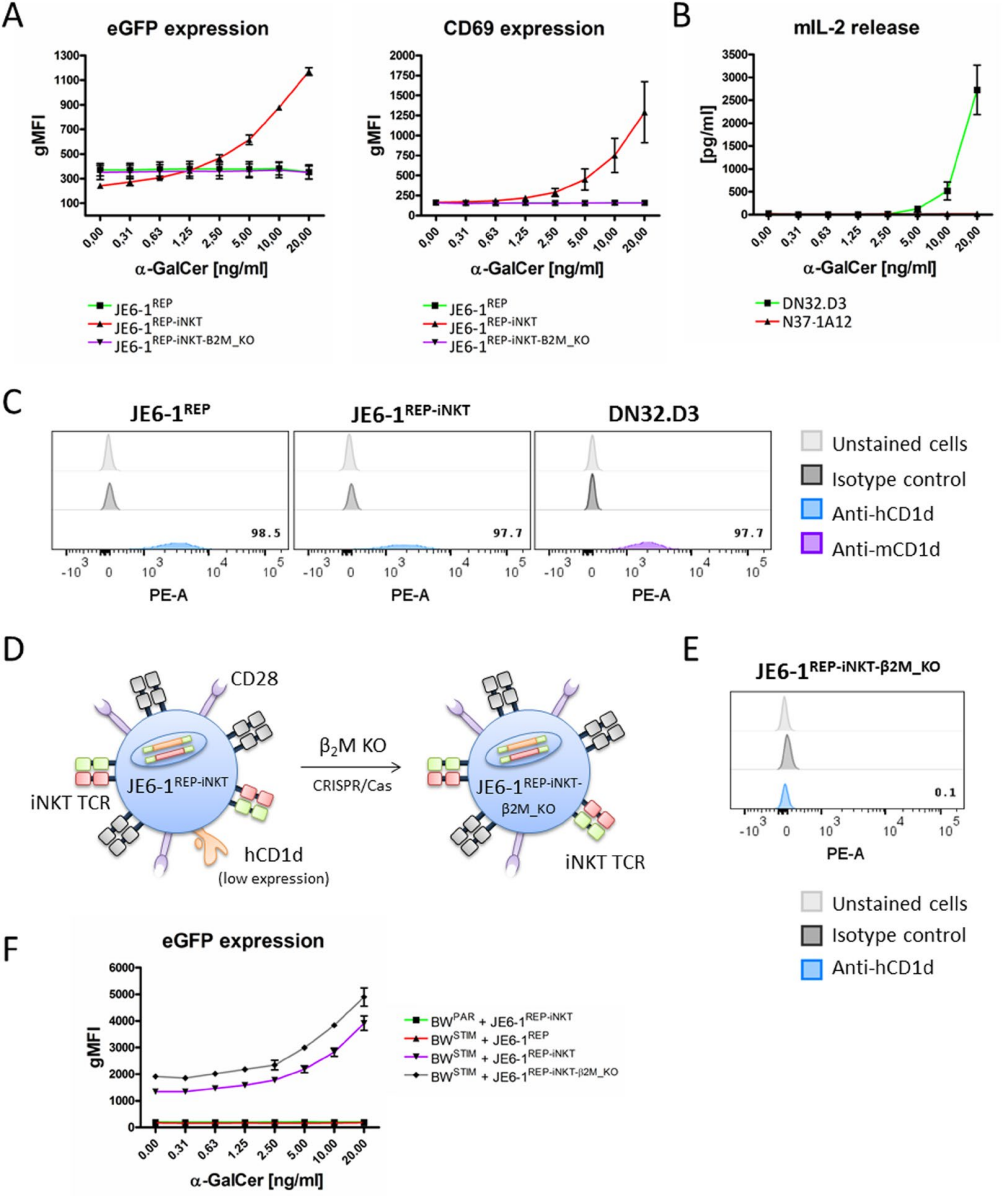
Knockout of β2 microglobulin (β2M) in JE6-1^REP-iNKT^ cells. (**A**) Activation of JE6-1 cells after overnight cultivation in the presence of soluble α-GalCer. Activation levels are represented as geometric mean of fluorescence intensity (gMFI) of eGFP (left) and CD69 (right). (**B**) Corresponding experiment to (A) using the murine hybridoma cell lines DN32.D3 and N37-1A12. Activation of murine hybridoma cells was assessed by IL-2 release. (**C**) CD1d surface staining of JE6-1^REP^, JE6-1^REP-iNKT^, and DN32.D3 cells. (**D**) β2M was genetically ablated using CRISPR/Cas9 in JE6-1^REP-iNKT^ cells. (**E**) CD1d surface staining of JE6-1^REP-iNKT-β2M_KO^ cells. (**F**) Comparison of JE6-1^REP-iNKT^ and JE6-1^REP-iNKT-β2M_KO^ cells in co-culture with the α-GalCer pulsed BW^STIM^ cell line. Activation levels are represented as geometric mean of fluorescence intensity (gMFI) of eGFP.

JE6-1^REP-iNKT^ cells constitutively show reporter activity upon addition of lipid antigen in the absence of antigen presenting cells as shown by upregulation of eGFP and CD69 (Fig. 5A). Dose-dependent activation was detectable starting at a concentration of 2.5 ng/ml α-GalCer. However, α-GalCer did not induce JE6-1^REP^ cell activation confirming that iNKT reporter cell activation is dependent on CD1d-iNKT-TCR interaction. Also the murine hybridoma cell line DN32.D3 secreted IL-2 (up to 3,500 pg/ml) after addition of α-GalCer in a dose-dependent manner. In contrast, the control hybridoma line N37-1A12 did not respond (Fig. 5B). JE6-1^REP^ and JE6-1^REP-iNKT^ cells as well as DN32.D3 constitutively express low levels of CD1d on their surface as shown by positive staining for human and murine CD1d, respectively (Fig. 5C). CD1d surface expression causes self-presentation of lipid antigens thus resulting in considerable reporter activity. In order to reduce T-T presentation we generated a β2 microglobulin knock out JE6-1^REP-iNKT^ cell line by CRISPR/Cas9, eliminating CD1d expression (Fig. 5D). The generated cells stained negative for CD1d (Fig. 5E) and did not display increased eGFP and CD69 expression upon addition α-GalCer (Fig. 5A). In co-cultures of α-GalCer pulsed BW^STIM^ cells, the sensitivity of JE6-1^REP-iNKT^ and JE6-1^REP-iNKT-β2M_KO^ was comparable as indicated by eGFP expression (Fig. 5F).

### Jurkat iNKT reporter cells can reveal antigenic properties of various lipid antigens

**Figure 6:**
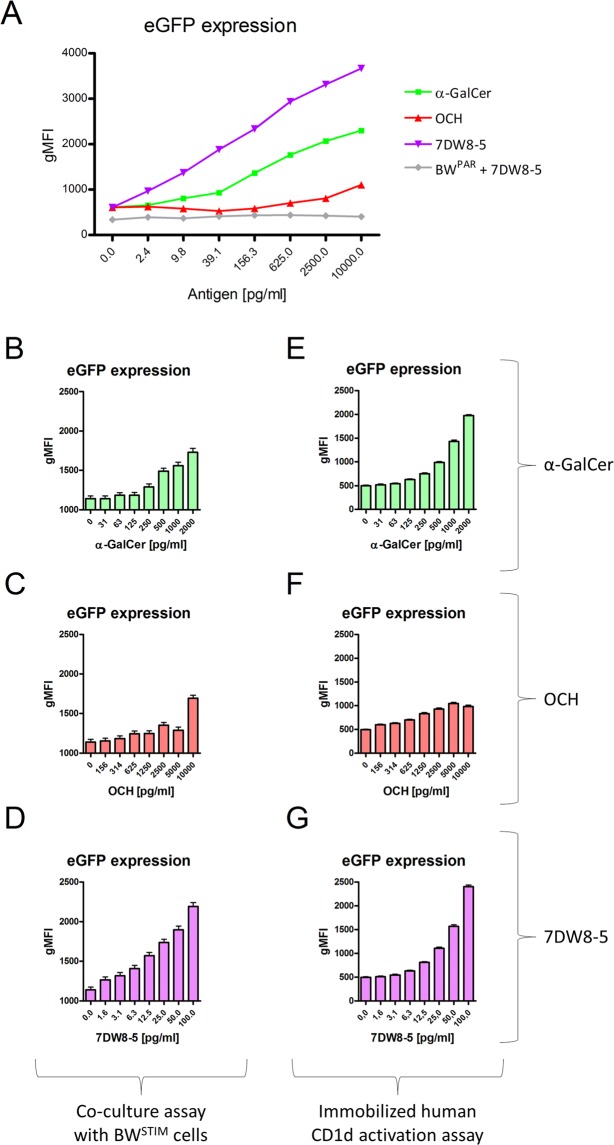
Capacity of different lipid antigens to activate JE6-1^REP-iNKT-β2M_KO^ reporters. Co-culture experiments of JE6-1^REP-iNKT-β2M_KO^ cells with BW^STIM^ cells performed in triplicates in the presence of selected lipid antigens. 7DW8-5 pulsed BW^PAR^ cells served as control (**A**). Analogous co-culture experiments with α-GalCer (**B**), OCH (**C**) and 7DW8-5 (**D**) using individual antigen concentration ranges. Cell activation using plate immobilized human CD1d monomers loaded with PBS diluted α-GalCer (**E**), OCH (**F**) and 7DW8-5 (**G**), respectively. Activation of reporter cells was determined via expression levels of eGFP and presented as geometric mean of fluorescence intensity (gMFI). (**B**–**G**) Three independent experiments performed in triplicates are shown.

We next wanted to assess whether our iNKT reporter cells can reveal differences in the stimulatory capacity of selected CD1d-presented lipid antigens. Figure 6A shows the dose-dependent activation capacities of α-GalCer and two derivatives, OCH and 7DW8-5, in co-culture assays with BW^STIM^ cells titrated up to 10 ng/ml. BW^PAR^ cells pulsed with 7DW8-5 served as negative control. 7DW8-5 induced activation of our reporter cells at the lowest concentration of 2.4 pg/ml. Cells responded in a dose-dependent manner without reaching a plateau. α-GalCer shows a similar activation curve with a reduced slope. In contrast, OCH, even at the highest concentrations, resulted in only very moderate stimulation (<30% of 7DW8-5 activation) of reporter cells. To examine the sensitivity of our JE6-1^REP-iNKT-β2M_KO^ reporter cells in more detail, we performed co-culture assays with BW^STIM^ cells (Fig. 6B–D), and antigen presentation assays with plate immobilized hCD1d (Fig. 6E–G). Concentration ranges of the different lipid antigens were adapted to their individual stimulation capacities in co-culture assays as presented in Fig. 6A. The same concentration ranges were used for both, BW^STIM^ cell pulsing and loading plate-bound hCD1d. In general, lower eGFP values were obtained with plate immobilized hCD1d (Fig. 6B–D) as compared to co-culture assays (Fig. 6E–G). JE6-1^REP-iNKT-β2M_KO^ responded to α-GalCer in a dose-dependent manner in both, co-culture and CD1d plate immobilized assays, with increased eGFP expression starting at 125 pg/ml. The highest concentrations (up to 10 ng/ml) were chosen for OCH (Fig. 6C,F) due to its lower stimulatory capacity. Although the slope was flat for OCH, differences in eGFP expression were still discernible and strictly dose-dependent. The lowest concentrations were applied for 7DW8-5 resulting in increased eGFP expression at 1.6 pg/ml and 6.3 pg/ml in co-culture and CD1d plate immobilized assay, respectively (Fig. 6D,G). Expression of eGFP was continuously increasing up to a concentration of 100 pg/ml without reaching a plateau of stimulation.

## Discussion

Invariant natural killer T (iNKT) cells are a distinct lineage of lymphocytes that are involved in the pathomechanisms of many different diseases while also contributing to homeostasis. For example, iNKT cells become activated during pathogen invasion, can suppress autoimmune diseases and allograft rejection and graft-versus host disease^40^. A better understanding of the diverse functions of iNKT cells is a prerequisite for the development of immunotherapeutic strategies targeting this cell lineage.

Invariant NKT cells can be grouped into different functional subsets based on characteristic surface molecules and highly divergent gene programs. Similar to conventional T helper cells, NKT1 (T-bet^+^ PLZF^lo^ RORγt^−^), NKT2 (PLZF^hi^ RORγt^−^ T-bet^−^), and NKT17 (RORγt^+^ T-bet^−^ PLZF^int^) cell populations were distinguished^41^. Moreover, the abundancy of these subsets is tissue dependent. For example, NKT1 cells are dominant in liver, spleen, lungs, thymus and small intestine of C57Bl/6 mice. In contrast, NKT17 cells are enriched in lymph nodes^13^. Moreover, cues from the local environment play an immanent role on the recruitment and stimulation of iNKT cells.

Primary human iNKT cells are difficult to obtain due to their low numbers in the peripheral blood. This hampers studies on these cells and makes it difficult to test candidate lipid antigens. In contrast to murine iNKT cells, human hybridoma cell lines are not available. To overcome these difficulties, we developed a modular human iNKT-TCR reporter cell system, using an already established Jurkat-based cell line equipped with an NF-κB::eGFP reporter construct^34^. Jurkat reporter cells stably transduced with the human iNKT-TCR (JE6-1^REP-iNKT^) specifically reacted with α-GalCer presented in the context of CD1d (plate bound assay) resulting in a dose-dependent activation as visualized by NF-κB-driven eGFP expression. When compared with the hybridoma cell line DN32.D3, our JE6-1^REP-iNKT^ cells showed equal sensitivity for α-GalCer. The main advantage of the reporter cell line is the possibility to directly measure NF-κB activation via eGFP expression without any additional staining procedures, making it a simple, fast and cost-effective system.

While certain lipids such as α-GalCer can directly be loaded onto CD1d, others require processing before being loaded and presented at the cell surface. To overcome this issue, we performed co-cultures with CD1d^+^ stimulator cells. JawsII, a professional murine APC line^42^, has been shown to highly express CD1d on their surface and to efficiently present α-GalCer^43^. When tested with our reporter cells, we found JawsII to function as APCs (data not shown). However, JawsII exhibit a slow growth rate and require addition of costly cytokines. We were therefore looking for an alternative APC cell line for our studies. Previously, we have used the rapidly dividing murine suspension cell line BW5147 to generate stimulator cells to activate primary T cells and human T cell reporters^44,45^. For the current study, we transduced the parental BW5147 with human CD1d and CD80 (BW^STIM^). BW^STIM^ are a convenient, easy-to-use, antigen presenting cell line, suitable for lipid antigen presentation via CD1d. We also compared BW^STIM^ to L-CD1d, an immortalized murine fibroblast cell line transduced with human CD1d^39^. L-CD1d cells express high amounts of CD1d on their surface loaded with agonistic endogenous lipid antigens and are able to stimulate iNKT cells even without addition of α-GalCer^46,47^. The advantage of this cell line is that this endogenous lipid antigen can be out-competed by other (endogenous or exogenous e.g. microbial) lipids that can either activate or inhibit our iNKT-reporter cells. Indeed, upon addition of increasing concentrations of α-GalCer, eGFP expression could eventually surpass the high background levels at concentrations above 250 pg/ml α-GalCer.

Low levels of CD1d expression in JE6-1^REP-iNKT^ cells led to self-presentation resulting in background activation. We could overcome this by knocking out β2M without affecting the sensitivity of the system. Because our reporter system is very easily transfected, it is well-suited for the study of genetic manipulations in TCR receptors and CD1d molecules. Our JE6-1^REP-iNKT-β2M_KO^ reporter cells can be used e.g. for investigations on the structural requirements of CD1d for binding of lipid antigens.

JE6-1^REP-iNKT-β2M_KO^ cells were activated with α-GalCer and the derivatives OCH and 7DW8-5, respectively. Differences in their stimulation capacity were consistent with the results of earlier studies^26^. This shows that our newly developed system is useful for testing a wide range of iNKT antigens.

Summarizing, we established a modular and convenient human iNKT reporter cell system to facilitate research on immunogenic iNKT lipid ligands. To date, α-GalCer, the iNKT prototypical antigen has been identified as a highly potent activator of iNKT cells and analogs of α-GalCer with a much stronger activation capacity are currently tested in clinical trials. However, knowledge of additional lipid antigens and their immunogenic activation capacity is limited. Utilizing the present iNKT reporter cell system as a high throughput screening tool could help to identify additional lipid candidates relevant for potential therapeutic applications. Moreover, further detailed studies are needed to address important questions such as the relevance of structural features of lipids and their binding affinity to the iNKT-TCR.

## Materials and Methods

### Cell culture

All cell culture media were supplemented with 10% FBS, 200 mM GlutaMAX, and penicillin/streptomycin unless otherwise stated. Jurkat and BW5147 based cell lines were cultured in RPMI 1640 medium. Murine iNKT cell hybridoma lines (DN32.D3 and N37-1A12) were cultured in Iscove’s Modified Dulbecco’s Medium supplemented with 5% FBS. The murine fibroblast cell line (L-cells) was kindly provided by Dr. Randy Brutkiewicz (Indiana University School of Medicine, Indianapolis, IN, USA) and cultured in DMEM supplemented with 500 μg/mL G418 for selection. All cell lines were cultivated at 37 °C in a humidified atmosphere (95%) containing 5% CO_2_.

### Flow cytometry

Mouse and human CD1d monomers, tetramers and dextramers (α-GalCer loaded/unloaded, APC-conjugated/Pacific-blue-conjugated/unconjugated) were kindly provided by the NIH Tetramer Core Facility (Atlanta, GA). Monoclonal anti-murine CD28-PE (37.51), CD1d-PE (CD1.1, Ly-38), CD80-PE (16-10A1), CD86-PE (GL-1), anti-human CD28-PE (CD28.2), CD1d-PE (51.1), CD80-PeCys7 (2D10), CD86-PeCys7 (IT2.2), and CD69-APC (FN50) antibodies were purchased from BioLegend (San Diego, CA). Anti-mouse CD45.2-PE (104) antibody was obtained from BD Bioscience (San Jose, CA). Flow cytometry analyses were performed using a BD FACSCanto II flow cytometer (BD Bioscience) and data were analyzed using the FlowJo software (version 10, Tree Star, Ashland, OR, USA).

### Generation of a Jurkat based NF-κB-eGFP iNKT reporter cell line

A human iNKT-TCR construct consisting of TCR beta (TCR V-beta chain: GenBank EAW51929.1) and alpha (TCR V-alpha chain: GenBank ABC72374.1) chains separated by a porcine teschovirus-1 2A (P2A) self-cleaving peptide sequence (iNKT-beta_P2A_alpha) was derived by gene synthesis (GeneArt, ThermoFisher, Heidelberg, Germany). The iNKT-beta_P2A_alpha fragment was cloned in frame into a modified lentiviral pHR‐SIN‐BX‐IRES‐Emerald vector, carrying a *Thosea asigna* virus 2A (T2A) sequence upstream of the puromycin N-acetyl-transferase (PAC) sequence, resulting in a single open reading frame consisting of iNKT-TCR beta, P2A, iNKT-TCR alpha, T2A and PAC (pHR-iNKT-TCR-2A-Puro). Jurkat E6.1 NF-κB::eGFP, a transcriptional reporter cells employing NF-κB-driven expression of eGFP^45^, were transduced with pHR-iNKT-TCR-2A-Puro and selected with puromycin at 1 μg/ml to obtain a uniformly iNKT-TCR positive cell population. Single cell clones were established from the transduced cell pool by limiting dilution and screened for their human iNKT-TCR expression using APC-conjugated CD1d tetramers. Highly expressing clones were chosen for functional tests on plate-bound α-GalCer loaded murine CD1d monomers (for details see below). A clone showing the strongest selective activation (designated JE6-1^REP-iNKT^) was used throughout the study.

### *In Vitro* Antigen Presentation Assay

MaxiSorp 96-well ELISA plates were coated with various concentrations (0.16–10.00 μg/ml) of α-GalCer loaded/unloaded murine/human CD1d monomers (kindly provided by NIH Tetramer Core Facility) together with the anti-CD28 antibody or isotype control overnight at 4 °C. For selected experiments, unloaded CD1d was coated and subsequently loaded with lipid antigens by overnight incubation with α-GalCer, OCH and 7DW8-5 diluted in PBS, respectively. Unspecific binding was blocked by addition of 5% fetal bovine serum (FBS) in PBS for two hours at room temperature. After washing with PBS, plates were incubated overnight with 5 × 10^4^ JE6-1^REP-iNKT^/murine hybridoma cells in 200 µl of the appropriate medium. After an overnight incubation at 37 °C cells were harvested, analyzed by flow cytometry and the geometric mean of fluorescence intensity (gMFI) was measured for each sample. Activation of murine hybridoma cells was analyzed by evaluating the IL-2 release after 24 hours of stimulation by ELISA according to the manufacturer’s protocol (BD Bioscience).

### Antigen self-presentation Assay

Reporter/murine hybridoma cells (5 × 10^4^) were seeded in 100 μl medium. Dilutions of α-GalCer were prepared and added to the wells to obtain final concentration range of 0–20 ng/ml. After 24 hours of incubation, cells were analyzed as described for the *in vitro* antigen presentation assay.

### Generation of BW based stimulator cell lines

Construction of the retroviral pCJK2-CD80 expression construct was described earlier^44^. Using the same strategy as for CD80, human CD1d was PCR amplified and cloned via SfiI into pCJK2. Murine thymoma cells BW5147 were simultaneously transduced with CD1d and CD80. A highly CD1d/CD80 double positive clone was derived by limiting dilution and used throughout the study.

### Co-culture assays with BW^STIM^ and L-CD1d cells

Stimulator cells (BW^PAR^/BW^STIM^/L-vector/L-CD1d, 5 × 10^5^) were incubated in conical tubes with various concentrations (1.6–2,000 pg/ml) of lipid antigens in the appropriate medium, respectively, for 4 hours at 37 °C in a water bath. After 3 washing steps with PBS, cells were resuspended in 1 ml of medium and seeded in 96-well u-bottom cell culture plates (100 µl/well). Reporter cells (JE6-1^REP^/JE6-1^REP-iNKT^/N37-1A12/DN32.D3) were added. After an overnight incubation at 37 °C cells were harvested and stained with an anti-mouse CD45 antibody and reporter cell activation was analyzed by FACS. Mouse CD45 positive cells (stimulator cells) were excluded from the analysis and the gMFI was measured for each sample. Median and standard deviation of triplicate wells were determined. For assays using hybridoma cell lines the supernatants were collected and the IL-2 was quantified by ELISA according to the manufacturer’s instruction (BD Bioscience).

### Knockout of β2 microglobulin via CRISPR/Cas9 technique

The 20nt single-guide RNA (sgRNA) was selected based on the β2 microglobulin (β2M) mRNA (NM_004048.2) by using the CRISPR design website (http://www.atum.bio). The target sequence 5′-GGCCGAGATGTCTCGCTCCG-3′ was inserted into the lentiCRISPR v2 vector purchased from Addgene (Cat. 52961; Cambridge, MA, USA) according to the protocol described by Zhang *et al*.^48,49^. In detail, the oligos (top: 5′-CACCG-20 nt; bottom: 5′-AAAC-20 nt) were annealed and cloned into the vector via BsmB1 sites. Correct insertion was confirmed by DNA sequencing using the sequencing primer 5′-GTACAAAATACGTGACG-3′. The resulting vector and virus packaging plasmids were transfected into HEK293T cells and cell culture supernatant containing lentiviral particles was used to transduce JE6-1^REP-iNKT^. After the selection with pyromycin, single cell clones were established. Genomic DNA from the selected clones was isolated (Gentra Purogene Cell Kit (Qiagen) and sequenced in a two-step PCR reaction described by Brinkmann *et al*.^50^. The sequence was then analyzed using the TIDE web tool (https://tide.deskgen.com) PMID: 25300484.
