# Publisher Correction: Generation of a Jurkat-based fluorescent reporter cell line to evaluate lipid antigen interaction with the human iNKT cell receptor

**DOI:** 10.1038/s41598-019-52827-w

**Published:** 2019-11-05

**Authors:** Piotr Humeniuk, Sabine Geiselhart, Claire Battin, Tonya Webb, Peter Steinberger, Wolfgang Paster, Karin Hoffmann-Sommergruber

**Affiliations:** 1 Department of Pathophysiology and Allergy Research, Medical University of Vienna, Vienna, Austria; 2 Institute of Immunology, Division of Immune Receptors and T cell Activation, Medical University of Vienna, Vienna, Austria; 3 Department of Microbiology & Immunology, University of Maryland School of Medicine, Baltimore, USA; 4 Present Address: Children’s Cancer Research Institute, St. Anna Kinderkrebsforschung, Vienna, Austria

Correction to: *Scientific Reports* 10.1038/s41598-019-43529-4, published online 15 May 2019

The original version of this Article contained a typographical error in the spelling of the author Karin Hoffmann-Sommergruber, which was incorrectly given as Karin Hoffmann-Sommergrsuber. This has now been corrected in the PDF and HTML versions of the Article.

